# P71 What are parents’ and patients’ perceptions of paediatric rheumatology shared-care in a London district general hospital? An evaluation to drive service improvement

**DOI:** 10.1093/rap/rkac067.071

**Published:** 2022-09-28

**Authors:** Lauren Huckerby, Shaniah Hussain, Nikita Thanki, Ashraf Gabr, John Ho

**Affiliations:** Department of Paediatrics, Whipps Cross University Hospital, Barts Health NHS Trust, London, United Kingdom; Department of Paediatrics, Whipps Cross University Hospital, Barts Health NHS Trust, London, United Kingdom; Department of Pharmacy, Whipps Cross University Hospital, Barts Health NHS Trust, London, United Kingdom; Department of Paediatrics, Whipps Cross University Hospital, Barts Health NHS Trust, London, United Kingdom; Department of Paediatrics, Whipps Cross University Hospital, Barts Health NHS Trust, London, United Kingdom

## Abstract

**Introduction/Background:**

Whipps Cross University Hospital is a District General Hospital (DGH) in London. Children with musculoskeletal and rheumatic conditions are looked after by a paediatric multidisciplinary team (MDT). In approximately one third of cases (around 50 patients), care is shared with tertiary teams.

It is widely cited in literature that patients with specialist conditions benefit from receiving specialist care. However, there are many positives of shared-care where patients also receive treatment in the local hospital. The provision of immunosuppressive treatment, regular blood monitoring, and appointments with MDT professionals (e.g. doctors/physiotherapists/ophthalmologists/community nurses) can add further complexities to shared-care.

**Description/Method:**

Our objective was to identify the strengths and weaknesses of the current Paediatric Rheumatology shared-care service at our DGH and to provide an action plan for improving the service.

Children and young people looked after by our DGH for a rheumatological condition, on immunosuppressants, whose care was shared with the tertiary centre were selected. Data collection was done via one-to-one telephone interviews with families based on a semi-structured questionnaire including the following four themes:

1. Views of shared care rheumatology.

2. Blood tests and the blood monitoring experience.

3. Medication related issues: obtaining, collecting and giving medication.

4. Views on transition to adult care.

The questionnaire was produced jointly by a paediatric doctor, the lead paediatric pharmacist and the lead nurse for the paediatric day unit, who is involved in blood tests, blood monitoring and delivering immunosuppressant medication. The questionnaire was piloted and modified. Interviews were conducted by a doctor not involved in the routine shared-care service to encourage honest answers, and to reduce observer and investigator bias. After data collection, quantitative and qualitative analysis (thematic analysis) were performed. Strengths and weaknesses of the service were identified.

**Discussion/Results:**

20 patients met our selection criteria and all 20 responded. Questionnaires were completed between April and July 2020.

On average, in the last year, patients visited their local DGH 2.5 times and their tertiary centre 2.9 times. 75% of these visits were outpatient appointments.

**Shared Care**

The following issues were identified by families through thematic analysis:

1. Proximity to home for appointments was important. The local team is responsive to their care.

2. Many appointments over both centres and missing school for appointments was a concern.

3. Local and tertiary centres don’t always share information.

4. Most families were happy to visit both hospitals.

**Blood Tests**

Blood testing was split between the GP, the local DGH, the tertiary hospital and community nurses. Blood tests were usually performed every 2-3 months. Families spoke positively about the blood testing experience. The suggested improvements were:

1. Proximity to home for blood tests to avoid missing school.

2. Better communication of results between hospitals.

3. A record of blood test results to keep at home.

**Medication-Related Issues**

70% of our cohort were taking methotrexate, and 20% were taking biologic medications. Prescriptions were provided by the GP, the local DGH and the tertiary centre.

Issues identified by families were:

1. Barriers to collecting medications (e.g. unhappy to leave the house due to COVID-19; unsure where to collect medication from; problems with obtaining prescriptions from the GP).

2. Better communication needed between hospitals about when new medication will start.

3. Patients would like to reduce the pain of the injection.

4. Methotrexate has too many side effects.

**Transitioning to Adult Care**

Most young people (60%) preferred to stay local to home and move to adult services at their local DGH, if there were to be an adolescent service with overlap between paediatric and adult services.

**Key learning points/Conclusion:**

The strengths and weaknesses of shared-care rheumatology were identified by families at our DGH. Strengths were that families felt both the local and tertiary centres played a necessary role in care. Weaknesses were that families had many appointments over both centres and missing school was problematic. Some families reported communication between hospitals could be improved.

The paediatric MDT proposed a list of quality improvement interventions:

1. Set up a one-stop Paediatric Rheumatology Super MDT clinic, to see the physiotherapist, ophthalmologist, paediatrician and pharmacist, if required, all in one morning. 2 one-stop clinics ran in March and May 2022. It is estimated that this super MDT clinic will save 3 to 4 DGH visits per year.

2. Obtain remote access to the tertiary hospital computer system for the local Paediatric Rheumatology team. This will improve communication between hospitals.

3. Encourage patients to view letters and results from the tertiary centre through the tertiary hospital patient app MyGOSH. This will improve timely information sharing.

4. Set up an MDT meeting with the day unit nurse and pharmacist every 2 months to discuss each patient requiring hospital prescribed medication.

5. The clinician to liaise with families around improving injection technique, and to discuss measures to mitigate side effects of medications.

6. Establish a system for posting medication to parents by pharmacy. This is in place as of August 2020 with an aim of reducing barriers to collecting medications.

7. Explore transitioning to alternative adolescent care closer to home.

Our next steps will involve re-assessing if the interventions above have helped improve communication with the tertiary hospital, whether they have reduced the number of appointments, and whether medication concerns were resolved. We believe that this innovative project has improved patient access to appointments, as well as their experience of the shared-care service.

